# Isolation and Multiple Differentiation Potential Assessment of Human Gingival Mesenchymal Stem Cells

**DOI:** 10.3390/ijms151120982

**Published:** 2014-11-14

**Authors:** Yuan Gao, Guizhi Zhao, Dongxia Li, Xin Chen, Jianliang Pang, Jie Ke

**Affiliations:** Department of Stomatology, Air Force General Hospital, People’s Liberation Army (PLA), Beijing 100142, China; E-Mails: lansefengqing1981@hotmail.com (Y.G.); guizhi1963@yeah.net (G.Z.); dongxia1973@126.com (D.L.); chenxin1959@126.com (X.C.); pangjian1977@126.com (J.P.)

**Keywords:** mesenchymal stem cells, gingiva, osteogenesis, adipogenesis, odontogenesis

## Abstract

The aim of this study was to isolate human mesenchymal stem cells (MSCs) from the gingiva (GMSCs) and confirm their multiple differentiation potentials, including the odontogenic lineage. GMSCs, periodontal ligament stem cells (PDLSCs) and dermal stem cells (DSCs) cultures were analyzed for cell shape, cell cycle, colony-forming unit-fibroblast (CFU-F) and stem cell markers. Cells were then induced for osteogenic and adipogenic differentiation and analyzed for differentiation markers (alkaline phosphatase (ALP) activity, mineralization nodule formation and Runx2, ALP, osteocalcin (OCN) and collagen I expressions for the osteogenic differentiation, and lipid vacuole formation and PPARγ-2 expression for the adipogenic differentiation). Besides, the odontogenic differentiation potential of GMSCs induced with embryonic tooth germ cell-conditioned medium (ETGC-CM) was observed. GMSCs, PDLSCs and DSCs were all stromal origin. PDLSCs showed much higher osteogenic differentiation ability but lower adipogenic differentiation potential than DSCs. GMSCs showed the medial osteogenic and adipogenic differentiation potentials between those of PDLSCs and DSCs. GMSCs were capable of expressing the odontogenic genes after ETGC-CM induction. This study provides evidence that GMSCs can be used in tissue engineering/regeneration protocols as an approachable stem cell source.

## 1. Introduction

Mesenchymal stem cells (MSCs) are multipotent stem cells that can be isolated from many tissues/organs, such as bone marrow and adipose tissue. With the properties of self-renewal and multi-lineage differentiation, MSCs are promising progenitor cell sources for stem cell transplantation, tissue engineering and regeneration [1]. Exploring suitable sources of stem cells for reparative and regenerative purposes is an important task in front of researchers.

Recent studies have confirmed the existence of MSCs from dental origin [2], providing potential cell sources for regeneration of tooth structures as well as other tissues/organs. The most widely known MSCs of dental origin are periodontal ligament stem cells (PDLSCs) [3]. Besides that, many other kinds of MSCs of dental origin have also been gradually isolated by the researchers, such as dental pulp stem cells (DPSCs) [4], stem cells from exfoliated deciduous teeth (SHED) [5], stem cells from the apical papilla (SCAP) [6], dental follicle stem cells (DFPCs) [7], *etc.* Though the ability of these cells to give rise to dental tissue as well some other tissues has been reported, unfortunately the accessibility and availability of these stem cells are quite limited. Comparatively, gingival MSCs (GMSCs) constitute more appealing alternatives to the other dental originated MSCs in terms of that they are much easier to get as a byproduct from the clinically resected gingival tissues. Hence, it is of great interest to confirm the multiple differentiation potentials of GMSCs for potential tissue engineering applications.

It is the aim of this study to systemically investigate the multiple differentiation abilities of GMSCs by comparing to PDLSCs, the most widely studied tooth originated MSCs, and dermal stem cells (DSCs), a kind of MSCs with similar origin to GMSCs. The data presented in this study provide evidence towards understanding the biological characteristics of GMSCs that could be used as a basis to support their superiority to the other MSCs for tissue engineering applications.

## 2. Results

### 2.1. Morphological and Cell Cycle Characteristics of the Primary Mesenchymal Stem Cells (MSCs) Established from Human Gingiva, Periodontal Ligament and Dermis

**Figure 1:**
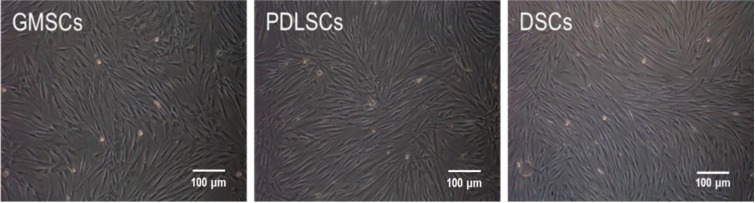
Morphology of the primary mesenchymal stem cells (MSCs) established from human gingiva, periodontal ligament and dermis (P3).

**Figure 2:**
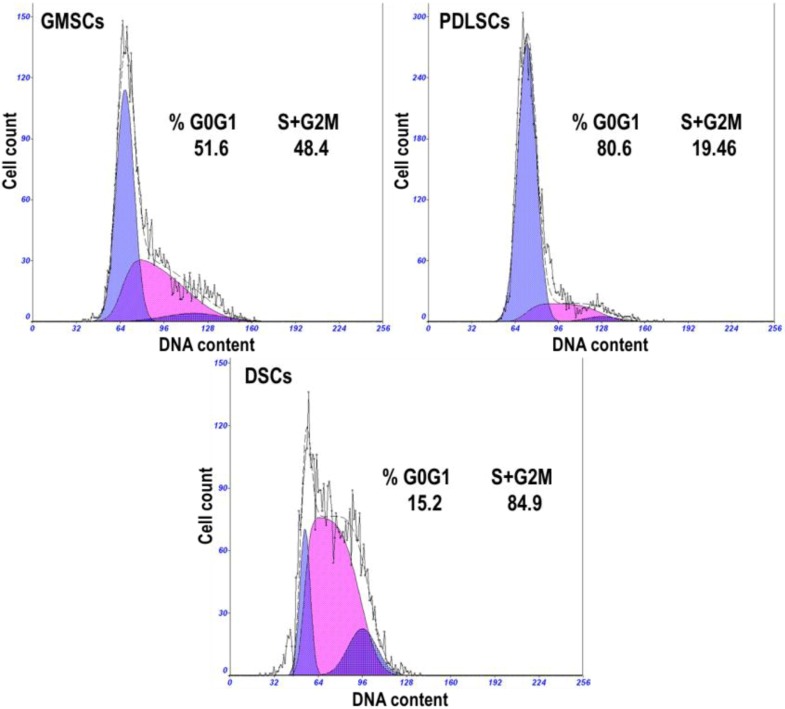
Representative cell cycle distribution graphs of gingival MSCs (GMSCs), periodontal ligament stem cells (PDLSCs) and dermal stem cells (DSCs) (P3). The percentages of cells residing in the G0/G1 phase, S and G2/M phases are shown in the graphs.

No significant differences were observed in the morphological characteristics of GMSCs, PDLSCs and DSCs (Figure 1). All the cells assumed a fibroblast-like spindle cell shape (Figure 1). The cell cycle propagation of MSCs was also monitored and the results are shown in Figure 2. GMSCs had more cells in the percentage of S + G2M phases (48.4%), compared to PDLSCs (19.46%), but lower when compared to DSCs (84.9%).

### 2.2. Colony-Forming Unit-Fibroblast Assay

**Figure 3:**
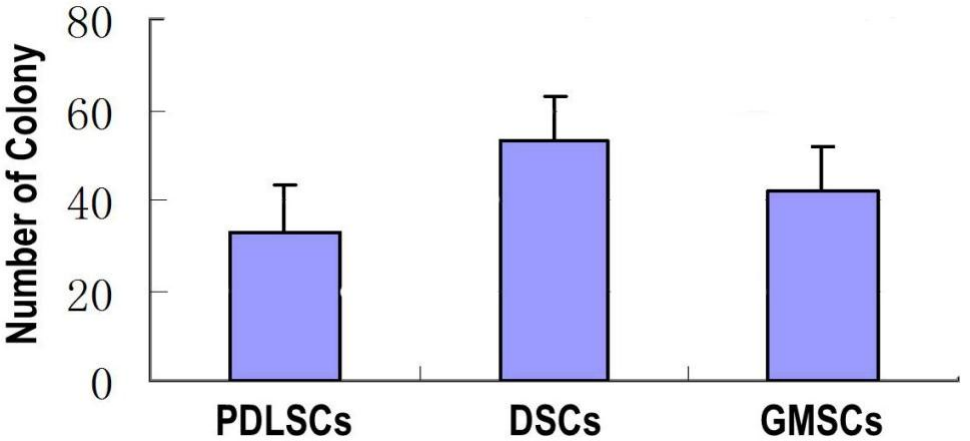
The number of colony generated by GMSCs, PDLSCs and DSCs (P3). The single P3 cell suspensions were seeded in 10 cm culture dishes in α-MEM (10% fetal bovine serum (FBS)) at a density of 1 × 10^3^ cells/well and cultured for 14 days.

The stem cells were characterized in terms of colony-forming unit-fibroblast (CFU-F) (Figure 3). Both types of cells displayed colony-forming ability, and the number of CFU-Fs for GMSCs was significantly higher than that of PDLSCs but lower than that of DSCs.

### 2.3. Immunophenotypic Characterization

**Figure 4:**
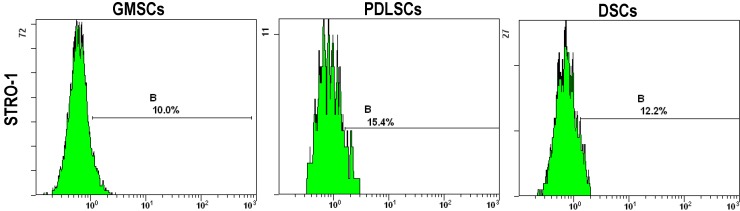

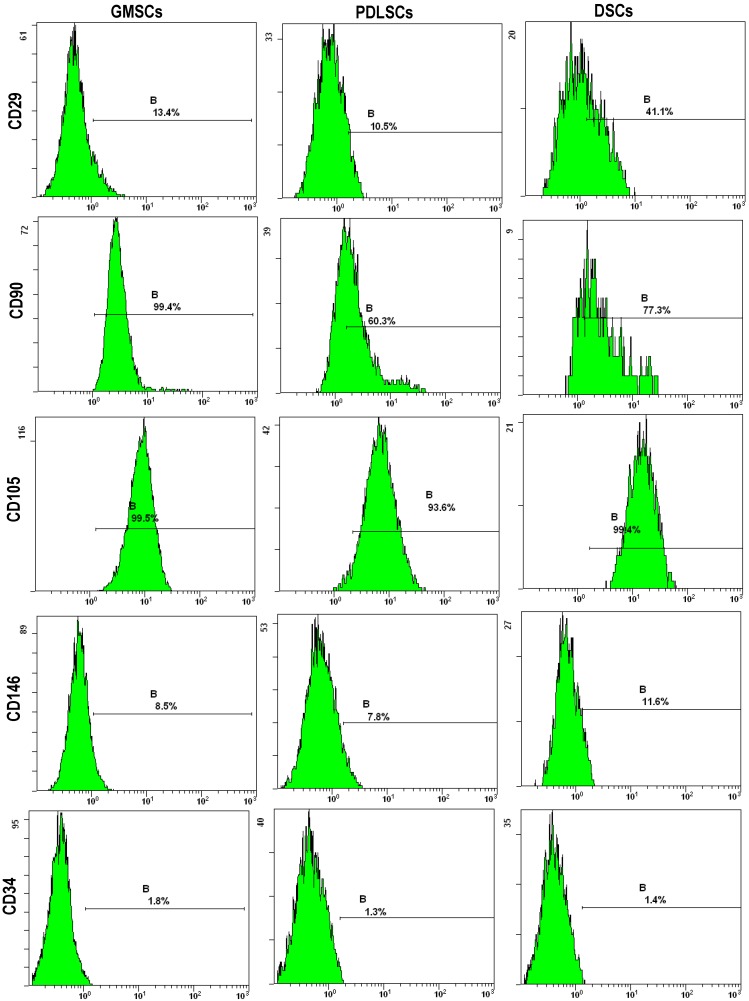

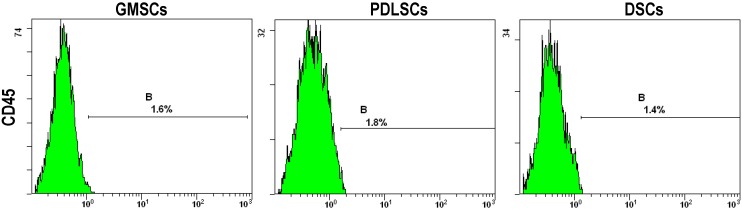
The expression of the stem cell surface markers including STRO-1, CD29, CD90, CD105 and CD146 and the leucocyte precursor markers CD45 and CD34 by GMSCs, PDLSCs and DSCs (P3). They were stained with antibodies for the markers and analyzed by flow cytometry. B indicates the expression levels of the surface markers.

Immunofluorescence analysis revealed that GMSCs, PDLSCs and DSCs cultures were positive for STRO-1, CD29, CD90, CD105 and CD146 that are the surface markers for MSCs (Figure 4 and Table 1). On the other hand, all types of cultures were negative for the leucocyte precursor markers CD45 or CD34, which suggests the stromal origin of the cells and the absence of hematopoietic precursor contamination. The quantitative data shown in Table 1 demonstrate that the three MSCs types showed very similar expression levels for the cell surface markers except that GMSCs expressed dramatically higher CD90 than PDLSCs and DSCs.

**Table 1:** The expression levels of the surface markers by GMSCs, PDLSCs and DSCs (P3) determined by flow cytometry.

	STRO-1	CD29	CD34	CD45	CD90	CD105	CD146
**GMSCs**	10.0	13.4	1.8	1.6	99.4	99.5	8.5
**PDLSCs**	15.4	10.5	1.3	1.8	60.3	93.6	7.8
**DSCs**	12.2	41.1	1.4	1.4	77.3	99.4	11.6

### 2.4. Osteogenic Differentiation Potential Assay

#### 2.4.1. Alkaline Phosphatase (ALP) Activity of GMSCs, PDLSCs and DSCs

**Figure 5:**
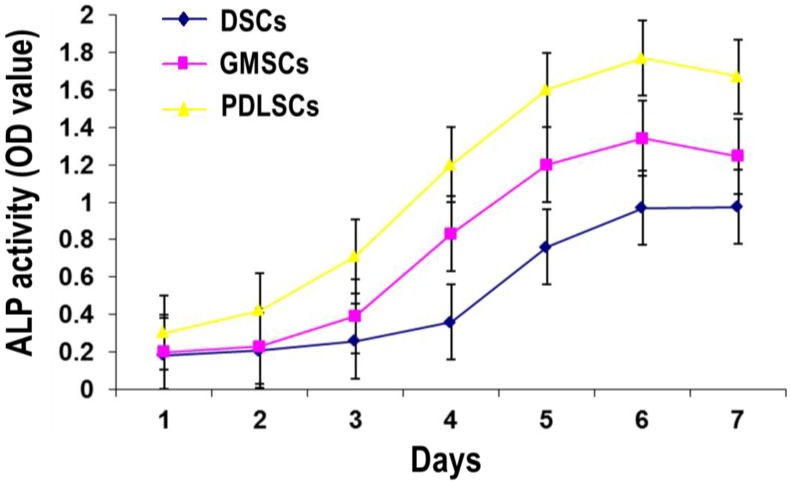
ALP activity of GMSCs, PDLSCs and DSCs (P3) after one to seven days of osteogenic induction.

The cells were cultured in the osteogenic medium for seven days, and ALP quantity was conducted (Figure 5). The results showed that for all the three cell types, the ALP activity firstly increased with time and peaked at day six. After that, the ALP activity declined a little. At all the time points, the ALP activity of the GMSCs was lower than that of PDLSCs but higher than that of DSCs.

#### 2.4.2. Mineralized Nodule Formation by GMSCs, PDLSCs and DSCs

**Figure 6:**
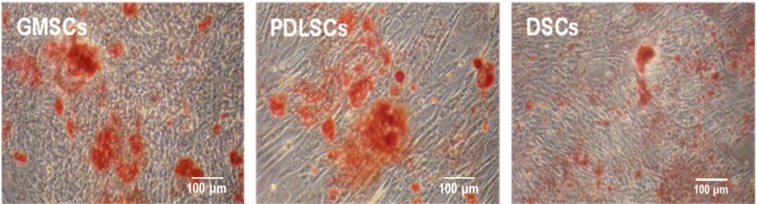
Mineralized nodule formation by GMSCs, PDLSCs and DSCs (P3) after three weeks of osteogenic induction.

When GMSCs, PDLSCs and DSCs were cultured in osteogenic medium for three weeks, all the cell types formed mineralized nodules as stained with Alizarin Red S (Figure 6). GMSCs seemed to generate similar level of mineralization to PDLSCs, which were both higher than that induced by DSCs.

#### 2.4.3. Osteogenic Gene Expression by GMSCs, PDLSCs and DSCs

**Figure 7:**
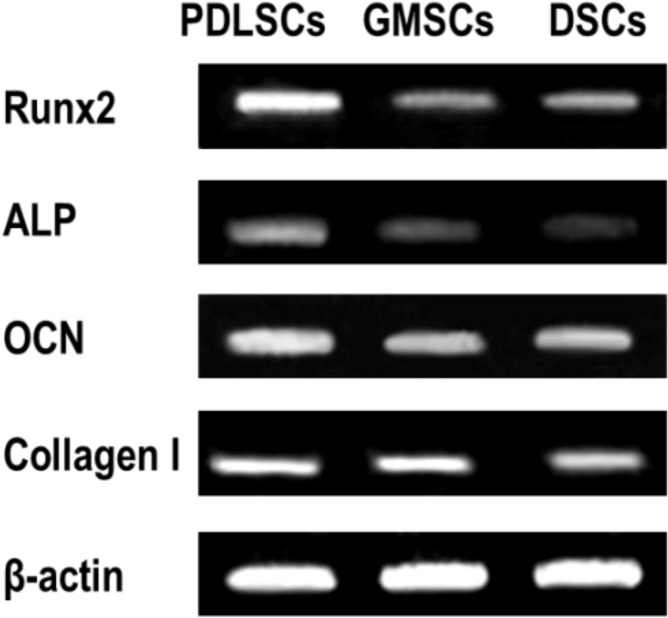
Expression of osteogenic genes including Runx2, ALP, OCN and collagen I by GMSCs, PDLSCs and DSCs (P3) after three weeks of osteogenic induction.

Reverse transcription polymerase chain reaction (RT-PCR) further indicated that the expression of the osteogenic genes including Runx2, ALP, osteocalcin (OCN) and collagen I by GMSCs was lower than that of PDLSCs. While, the expression of ALP, OCN and collagen I by GMSCs was higher than that of DSCs (Figure 7).

### 2.5. Adipogenic Differentiation Potential Assay

**Figure 8:**
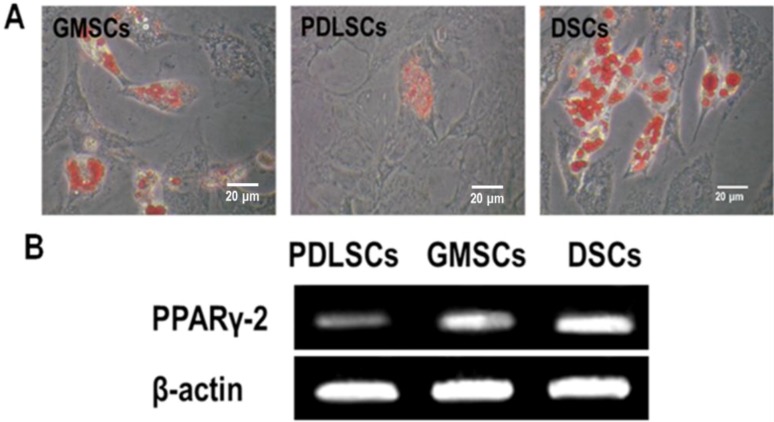
Adipogenic differentiation of GMSCs, PDLSCs and DSCs (P3) after four weeks of adipogenic induction. (**A**) Representative images of intracellular lipid vacuoles that appeared in all tested cells, as confirmed by Oil Red O staining; and (**B**) The expression of an adipogenic gene PPARγ detected by RT-PCR.

When GMSCs, PDLSCs and DSCs were cultured in adipogenic medium for four weeks, all the cell types formed lipid vacuoles as confirmed by Oil Red O staining (Figure 8A). Being opposite to the trend of osteogenic differentiation, DSCs generated the largest amounts of lipid vacuoles, followed by GMSCs and PDLSCs generated the least. This was further demonstrated by analyzing the mRNA levels of PPARγ-2, which is an adipocyte-specific transcript (Figure 8B).

### 2.6. Odontogenic Differentiation Potential of GMSCs

**Figure 9:**
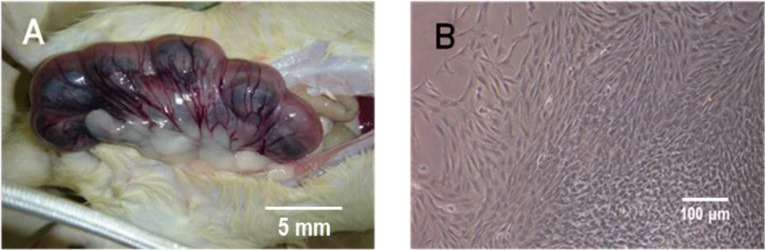

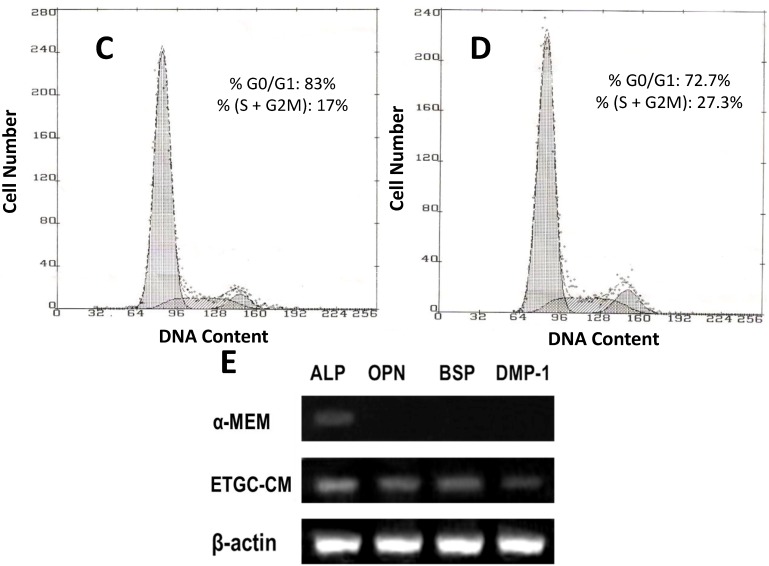
Odontogenic differentiation of GMSCs after two weeks of induction. (**A**) Representative images of embryonic Sprague-Dawley rats; (**B**) Representative images of tooth germ cells (TGCs). Representative cell cycle distribution graphs of GMSCs cultured with α-MEM (**C**) and ETGC-CM (**D**) for eight days; and (**E**) The expression of odontogenic genes detected by RT-PCR.

The cell cycle propagation of GMSCs without or with the embryonic tooth germ cell-conditioned medium (ETGC-CM) induction was shown in Figure 9C,D. The ETGC-CM induced GMSCs had more cells in the percentage of S + G2M phases (27.3%) compared to the non-induced ones (17%), indicating that the ETGC-CM induction can promote the proliferation of GMSCs. The RT-PCR results demonstrated that the ETGC-CM induction led to obviously higher expression levels of odontogenic genes including ALP, osteopontin (OPN), bone sialoprotein (BSP) and dentin matrix protein 1 (DMP-1) (Figure 9E).

## 3. Discussion

Gingival tissue plays an important role by acting as a barrier against different insults, such as chemicals and bacteria, and it exhibits a unique scarless healing process after wounding instead of the scar formation [8], suggesting that unique types of MSCs reside in gingival tissues. Zhang *et al.* firstly isolated a population of progenitor/stromal cells within gingival tissues that show stem cell nature [9]. GMSCs constitute more appealing alternatives to the other dental originated MSCs in terms of that they are much easier to get as a byproduct from the clinically resected gingival tissues. Herein to demonstrate the suitability of GMSCs for future applications in regeneration protocols of different purposes, the multiple differentiation potentials of GMSCs towards osteo/adipogenic phenotypes were compared to those of PDLSCs, the most widely known MSCs of dental origin, and DSCs, a kind of MSCs with similar origin to GMSCs. The odontogenic differentiation of GMSCs was also inspected. GMSCs were stromal origin, they possessed higher proliferative ability, colony-forming ability and adipogenic differentiation ability but relatively lower osteogenic differentiation ability compared to PDLSCs. In addition, GMSCs could differentiate toward the odontogenic line under the stimulation of ETGC-CM. Our study shall provide novel insight to the potential application of GMSCs for regenerative purposes.

The morphology of GMSCs showed no discernible difference compared to that of PDLSCs and DSCs. They all showed fibroblastic cell morphology, the typical shape of MSCs. While the analysis of their growth characteristics showed notable differences among the three cell types. GMSCs had more cells in the percentage of S + G2M phases (48.4%) compared to PDLSCs (19.46%), which is in accordance with several previous studies that GMSCs have a higher rate of successive culture and proliferation compared with PDLSCs [10,11]. However, GMSCs showed lower percentage of S + G2M phases compared to DSCs (84.9%). The colony forming efficiency of the three MSCs types follows the trend of DSCs > GMSCs > PDLSCs, which is well in line with the cell cycle analysis results. The higher proliferative and colony forming abilities of DSCs and GMSCs are also well in accordance with the fact that the skin and the gingiva have higher healing and regenerative abilities than the periodontal ligament. The higher proliferative and colony forming abilities for GMSCs are beneficial for the regenerative applications in terms of easy *in vitro* duplication as well as finally massive tissue regeneration.

The analysis of stem cell surface epitopes revealed no differences in the immunophenotypic profiles for GMSCs, PDLSCs and DSCs. They were positive for STRO-1, CD29, CD90, CD105 and CD146 that are the surface markers for MSCs and were negative for the leucocyte precursor markers CD45 or CD34. The immunophenotypic profiles further verified the stromal origin of our cell culture without hematopoietic precursor contamination. It is interesting that compared to PDLSCs and DSCs, GMSCs expressed dramatically higher CD90. Hsu *et al.* also reported a high expression of CD90 by the gingival fibroblasts [12], which is in line with our results.

Then we have made a systemic comparison on the osteogenic and adiopogenic differentiation potentials of GMSCs, PDLSCs and DSCs. Interestingly, PDLSCs showed much higher osteogenic differentiation ability but lower adipogenic differentiation potential than DSCs. GMSCs showed the medial osteogenic and adipogenic differentiation potentials between those of PDLSCs and DSCs. To our knowledge, our present study has made the first comparison on the multiple differentiation abilities of GMSCs, PDLSCs and DSCs. GMSCs that also show the differentiation abilities to bone lineage are a good candidate stem cells for bone regeneration. Actually, GMSCs are found to be superior to BMSCs for cell therapy in bone regenerative medicine [13].

Finally, we have confirmed the odontogenic differential of GMSCs under the induction of ETGC-CM. ETGC-CM can provide the environment for odontogenic differentiation of stem cells and is widely used in studying the odontogenic differentiation potential of MSCs [14]. Herein, our results demonstrated that GMSCs were capable of expressing the odontogenic genes including ALP, OPN, BSP and DMP-1 after ETGC-CM induction, explicitly demonstrating the potential of GMSCs towards the odontogenic lineage. To our knowledge, this is the first evidence for the odontogenic potential of GMSCs. Recently, there are several reports indicating that GMSCs show broad application potential and can lead to possible regeneration of bone tissue [15], periodontal tissue and tendon tissue [16,17]. Our data further expand the potential applications for GMSCs.

## 4. Experimental Section

### 4.1. Isolation of Primary MSCs from Human Gingiva, Periodontal Ligament and Dermis

Gingival tissues and teeth were obtained from healthy patients (18–35 years old) undergoing third molar extraction. Biopsy samples from foreskin were taken for diagnostic purposes from people aged 10–18 years, and all the samples used were macroscopically normal. The use of human gingival tissues, teeth and skin for research purpose was approved by the Institutional Review Board (IRB) of The Fourth Military Medical University, School of Stomatology. The informed consent was obtained from each subject. Human PDLSCs, GMSCs and DSCs were isolated and cultured according to previously published procedures [3,9]. Briefly, to get PDLSCs, after rinsing with phosphate buffered saline (PBS, Sigma–Aldrich, St. Louis, MO, USA), and periodontal ligament tissues on the middle third of the tooth root were separated, collected and digested with 3 mg/mL type I collagenase (Sigma–Aldrich) and 2 mg/mL dispase (Sigma–Aldrich) at 37 °C for 15 min. The tissues were then transferred to six-well culture dishes (Corning, Corning, NY, USA) and cultured in α-MEM supplemented with 10% FBS (Sigma–Aldrich) at 37 °C in a humidified atmosphere with 5% CO_2_ to allow the cells grow out from the tissue patch. To obtain GMSCs or DSCs, the gingival or skin tissues were treated aseptically and incubated overnight at 4 °C with 2 mg/mL dispase to separate the epithelial and the spinous layers. The tissues were minced into fragments and digested with 4 mg/mL collagenase IV (Worthington Biochemical, Lakewood, NJ, USA) at 37 °C for 2 h. The dissociated cell suspension was filtered through a 70 μm cell strainer (Falcon, Heidelberg, Germany), plated on 10 cm petri dishes (Corning) with α-MEM supplemented with 10% fetal bovine serum. For all the cell types, three days later the nonadherent cells were removed and the culture medium was refreshed every 3 days.

### 4.2. Single Cell Cloning

Single cell cloning was realized by limiting dilution method. Succinctly, suspensions of the primary cells were diluted to a density of 10 cells/mL, and 100 μL of the cells suspensions was seeded in a 96-well plate. The wells containing a single cell were selected microscopically, and after reaching 80% confluence they were harvested and amplified. These cells were marked as passage 1 (P1). Cells at passages P3–P4 were used in the present study.

### 4.3. Colony-Forming Unit-Fibroblast Assay

To assess the colony forming efficiency of the three kinds of MSCs, the single P3 cell suspensions were seeded in 10 cm culture dishes in α-MEM (10% FBS) at a density of 1 × 10^3^ cells/well and cultured for 14 days. The cell cultures were fixed in 4% paraformaldehyde, stained with 1% toluidine blue, washed with distilled water, and dried. Aggregates of more than 50 cells were scored as a CFU-F colony.

### 4.4. Analysis of Surface Marker Expression

The MSCs of different origins (P3) were stained with antibodies for stem cell surface markers and analyzed by flow cytometry. Briefly, to identify the phenotypes of the MSCs, P3 cells of 5 × 10^5^ were incubated with fluorescein isothiocynante (FITC) conjugated monoclonal antibodies for human STRO-1 (Biolegend, San Diego, CA, USA), CD29 (eBioscience, San Diego, CA, USA) and phycoerythrin (PE) conjugated monoclonal antibodies for human CD146 (Biolegend), CD34 (Biolegend), CD105 (eBioscience), CD90 (eBioscience) and CD45 (Abcam, Cambridge, UK), as the manufacturer’s instructions. The incubation procedure was carried out at 4 °C away from light for 1 h. After thorough washing with PBS, the cells were subjected to flow cytometric analysis (Beckman Coulter, Fullerton, CA, USA).

### 4.5. Cell Cycle Analysis

The MSCs of different origins were trypsinized and pooled for cell cycle analysis. The cells were washed with PBS and resuspended in 1 mL of PBS by repeated vibration to get a suspension. Then the cells in the suspension were fixed with ice-cold dehydrated ethanol overnight at 4 °C. The fixed cells were washed twice with PBS and stained by 100 mg/mL propidium iodide (PI, Sigma–Aldrich) at 4 °C for 30 min. The PI-elicited fluorescence of individual cells was measured using Elite ESP flow cytometry (FCM, Beckman Coulter). At least 4 × 10^4^ cells were analyzed for each group. The amounts of cells residing in the G0/G1 phase, S phase, and G2/M phase were determined.

### 4.6. Osteogenic Differentiation Induction

For the induction of osteogenic differentiation, the MSCs were seeded in 6 well plates at a density of 2 × 10^4^/cm^2^. After the cells reaching 80% confluence, they were induced for osteogenic differentiation by being exposed to a osteogenic medium by adding osteogenic supplement comprising 100 nM dexamethasone, 10 mM β-glycerophosphate and 50 μg/mL ascorbic acid to the α-MEM (10% FBS) (all from Sigma–Aldrich). The cells were cultured for a total of 3 weeks with the osteogenic medium being changed every 3–4 days. The cell cultures were processed for ALP activity assay and RT-PCR analysis and evaluated for mineralization by Alizarin Red S (AR-S) staining.

#### 4.6.1. Alkaline Phosphatase Activity Assay

After 1–7 days of osteogenic induction, the cells were washed with PBS and lysed in 0.1 vol% Triton X-100 through freeze-thaw. The ALP activity in the lysis was determined by means of a colorimetric assay using an ALP reagent containing p-nitrophenyl phosphate (p-NPP, Jiancheng Bioengineering Institute, Nanjing, China). The absorbance of the formed p-nitrophenol was measured at 405 nm. The intracellular total protein content was determined using the MicroBCA protein assay kit (Beyotime, Shanghai, China) and the ALP activity was normalized to it.

#### 4.6.2. Mineralization Assay

After 3 weeks of osteogenic induction, extracellular matrix (ECM) mineralization was evaluated by AR-S staining. The cells were washed twice with PBS and fixed with 75% ethanol for 1 h. Then, cells were stained with 1% AR-S (Sigma–Aldrich) (pH 4.2) for 20 min at RT. Cells were then rinsed three times with distilled water to reduce non-specific staining. Mineralized nodules were visualized and photographed using an inverted microscope (Olympus Optical Co., Ltd., Tokyo, Japan) and the area covered with mineralized tissue was determined.

#### 4.6.3. RT-PCR Analysis on Osteogenic Gene Expression

After 3 weeks of osteogenic induction, the total RNA was isolated using TRIzol (Gibco BRL, Gaithersburg, MD, USA), which was reverse transcribed into complementary DNA (cDNA) using PrimeScript™ RT (TaKaRa, Dalian, China). Gene product amplification was performed in a TGradient thermocycler (Biometra, Göttingen, Germany). PCR-amplified fragments were resolved by 1.5%–3.0% agarose/ethidium bromide gel electrophoresis. Bands were analyzed using a GDS-8000 Gel analysis system (UVP) combinated with Image J software (Version 1.36b for Windows, National Institutes of Health, Maryland, USA). Each value is expressed as arbitrary densitometric units normalized against values for β-actin. The primers for the target genes were listed as follows: Runx2 (sense: 5'-CACTGGCGCTGCAACAAGA-3', antisense: 5'-CATTCCGGAGCTCAGCAGAATAA-3'), ALP (sense: 5'-GGACCATTCCCACGTCTTCAC-3′, antisense: 5'-CCTTGTAGCCAGGCCCATTG-3'), OCN (sense: 5'-CCCAGGCGCTACCTGTATCAA-3', antisense: 5'-GGTCAGCCAACTCGTCACAGTC-3'), collagen I (sense: 5'-CCAGAAGAACTGGTACATCAGCAA-3', antisense: 5'-CGCCATACTCGAACTGGAATC-3') and β-actin (sense: 5'-TGGCACCCAGCACAATGAA-3', antisense: 5'-CTAAGTCATAGTCCGCCTAGAAGCA-3') as housekeeping control gene.

### 4.7. Adipogenic Differentiation Induction

For the induction of adipogenic differentiation, the MSCs were seeded in 6 well plates at a density of 2 × 10^4^/cm^2^. After the cells reaching 80% confluence, they were exposed to an adipogenic medium by adding adipogenic supplement comprising 0.5 mM methylisobutylxanthine, 0.5 mM hydrocortisone, and 60 mM indomethacin to the α-MEM (10% FBS) (all from Sigma–Aldrich). The cells were cultured for a total of 4 weeks and were then processed for RT-PCR analysis and evaluated for intracellular lipid accumulation by staining with oil red O (Sigma–Aldrich).

#### 4.7.1. Oil Red O Staining

The cells were washed three times in PBS after fixation in 4% paraformaldehyde for 10 min and then incubated in 0.3% oil red O solution for 15 min. After being washed with PBS, the cells were observed and photographed under a phase-contrast inverted microscope.

#### 4.7.2. RT-PCR Analysis on Adipogenic Gene Expression

At the prescribed time point, the RT-PCR was conducted same as described in 2.6.3. The primers for the target genes were listed as follows: PPARγ (sense: 5'-CCGTGGTCTCTCCGTAATG-3', antisense: 5'-ACTCTGGATTGAGCTGGTCG-3') and β-actin (sense: 5'-TGGCACCCAGCACAATGAA-3', antisense: 5'-CTAAGTCATAGTCCGCCTAGAAGCA-3') as housekeeping control gene.

### 4.8. Odontogenic Differentiation Potential of GMSCs

The ETGC-CM was prepared as follows. The tooth germs were isolated from the lower molars of embryonic Sprague-Dawley rats and minced into less than 1 mm^3^ pieces in PBS. After digestion with type I collagenase (Gibco BRL) for 40–60 min at 37 °C. The TGCs were collected by centrifugation and washed twice with PBS. Single cell suspensions were obtained by filtration through a 70 μm strainer and were then washed with PBS. The cells were then placed into 75 cm^2^ culture flasks at a concentration of 1 × 10^5^ cells/mL and grown in 5% CO_2_ at 37 °C. The culture medium for the primary TGCs containing both epithelial and mesenchymal cells was changed every day. Once reaching 100 confluence, the supernatant was collected and filtered with 0.22 μm strainer and mixed with an equal volume of fresh α-MEM (10% FBS), which was used as ETGC-CM.

For the induction of odontogenic differentiation, GMSCs were seeded in 6 well plates at a density of 2 × 10^4^/cm^2^. After the cells reaching 80% confluence, they were induced for osteogenic differentiation by being exposed to ETGC-CM. The cells cultured with α-MEM (10% FBS) served as control. After 8 days of induction, the cell cycle analysis was conducted. After 10 days of induction, the expression of odontogenic genes was analyzed by RT-PCR and the primers for the target genes were listed as follows: OPN (sense: 5'-AGGACTCCATTGACTCGAACGA-3', antisense: 5'-CACCTCGGCCATCATATGTGT-3'), BSP (sense: 5'-CTGGCACAGGGTATACAGGGTTAG-3', antisense: 5'-ACTGGTGCCGTTTATGCCTTG-3'), and DMP-1 (sense: 5'-TTGTCACATAAAGGAGTCTTAGGGA-3', antisense: 5'-ACCTGCTTTACATTCTCAACACCTA-3').

### 4.9. Statistical Analysis

All the assays were performed on at least three independent experiments (*n* = 3), with 3 replicates each and the results were expressed as means ± standard deviations (SD). One way ANOVA combined with Student-Newmane-Keuls *post hoc* test was used to determine the statistical significance among different groups (significance assumed for *p* < 0.05).

## 5. Conclusions

GMSCs of stromal origin can be successfully isolated from the human gingival tissue and cultured *in vitro*. GMSCs possessed higher proliferative ability, colony-forming ability and adipogenic differentiation ability but relatively lower osteogenic differentiation ability compared to PDLSCs. GMSCs also showed the differentiation ability to the odontogenic lineage under suitable stimulation. Our data suggest that GMSCs may constitute a good candidate for regeneration of various tissues.
